# Screening of xylose utilizing and high lipid producing yeast strains as a potential candidate for industrial application

**DOI:** 10.1186/s12866-022-02586-y

**Published:** 2022-07-07

**Authors:** Linnea Qvirist, Friederike Mierke, Ricardo Vazquez Juarez, Thomas Andlid

**Affiliations:** 1 Department of Biology and Biological Engineering, Food and Nutrition Science, Chalmers University of Technology, SE-412 96 Gothenburg, Sweden; 2 Department of Biology and Biological Engineering, Systems and Synthetic Biology, Chalmers University of Technology, SE-412 96 Gothenburg, Sweden; 3 Laboratorio de genomica, CIBNOR, 23096 La Paz, Mexico

**Keywords:** Microbial lipids, Oleaginous yeast, Lignocellulose, Pseudozyma hubeiensis, *Rhodosporidium toruloides*

## Abstract

**Background:**

Sustainable production of oil for food, feed, fuels and other lipid-based chemicals is essential to meet the demand of the increasing human population. Consequently, novel and sustainable resources such as lignocellulosic hydrolysates and processes involving these must be explored. In this paper we screened for naturally-occurring xylose utilizing oleaginous yeasts as cell factories for lipid production, since pentose sugar catabolism plays a major role in efficient utilization of lignocellulosic feedstocks. Glycerol utilization, which is also beneficial in yeast-based oil production as glycerol is a common by-product of biodiesel production, was investigated as well. Natural yeast isolates were studied for lipid accumulation on a variety of substrates, and the highest lipid accumulating strains were further investigated in shake flask cultivations and fermenter studies on xylose and hydrolysate.

**Results:**

By collecting leaves from exotic plants in greenhouses and selective cultivation on xylose, a high frequency of oleaginous yeasts was obtained (> 40%). Different cultivation conditions lead to differences in fatty acid contents and compositions, resulting in a set of strains that can be used to select candidate production strains for different purposes. In this study, the most prominent strains were identified as *Pseudozyma hubeiensis* BOT-O and *Rhodosporidium toruloides* BOT-A2. The fatty acid levels per cell dry weight after cultivation in a nitrogen limited medium with either glucose, xylose or glycerol as carbon source, respectively, were 46.8, 43.2 and 38.9% for *P. hubeiensis* BOT-O, and 40.4, 27.3 and 42.1% for BOT-A2. Furthermore, BOT-A2 accumulated 45.1% fatty acids per cell dry weight in a natural plant hydrolysate, and *P. hubeiensis* BOT-O showed simultaneous glucose and xylose consumption with similar growth rates on both carbon sources. The fatty acid analysis demonstrated both long chain and poly-unsaturated fatty acids, depending on strain and medium.

**Conclusions:**

We found various natural yeast isolates with high lipid production capabilities and the ability to grow not only on glucose, but also xylose, glycerol and natural plant hydrolysate. *R. toruloides* BOT-A2 and *P. hubeiensis* BOT-O specifically showed great potential as production strains with high levels of storage lipids and comparable growth to that on glucose on various other substrates, especially compared to currently used lipid production strains. In BOT-O, glucose repression was not detected, making it particularly desirable for utilization of plant waste hydrolysates. Furthermore, the isolated strains were shown to produce oils with fatty acid profiles similar to that of various plant oils, making them interesting for future applications in fuel, food or feed production.

**Supplementary Information:**

The online version contains supplementary material available at 10.1186/s12866-022-02586-y.

## Background

Oleaginous yeasts (oil accumulating) are increasingly being explored as an alternative for production of nutritional supplements in food and feed, as well as other lipid related biochemicals. The utilization of yeasts, rather than plants, for lipid production has many advantages: yeasts are fast-growing microorganisms that are independent of season and climate, have less space requirements, minimize competition with food production, and their cultivation does not require the use of pesticides.

To achieve competitive lipid production using oleaginous yeasts, the following traits are beneficial: i) robustness in a sustainable substrate such as industry waste or side streams, ii) high lipid production per biomass unit, carbon source and time, iii) ability to efficiently utilize pentoses, such as xylose, to achieve higher overall lipid yield from lignocellulose substrates, and iv) ability to produce lipids from glycerol, which is a by-product when converting storage lipids to biodiesel (roughly 100 kg per ton of fatty acid methylesters [1]).

The fatty acid (FA) composition of yeast oils varies between species and strains, but the most common FA profiles resemble plant oils [2, 3], such as olive and canola oil, with high relative content of C18:1. Generally, the longer and more unsaturated fatty acids occur in lower amounts, with the exception of long chain polyunsaturated fatty acids (PUFA), e.g. C20:5 and C22:6, which are associated with several health benefits [4]. *Yarrowia lipolytica* has been successfully modified to produce increased amounts of PUFA [5, 6] but is not naturally able to utilize xylose as a carbon source [7].

Only a small fraction of the known yeast species are oleaginous, and only about 5% of those have been reported to accumulate over 25% lipids [8]. Species known to accumulate lipids include *Trichosporon asahii, Cutaneotrichosporon oleaginosus, Y. lipolytica,* as well as the red yeasts *Rhodotorula glutinis, Rhodotorula mucilaginosa, Rhodosporidium toruloides,* and some *Candida* species [2, 8, 9]. Both *R. glutinis* and *R. toruloides* are well-studied yeasts able to utilize pentose sugars [10–14]. They have been isolated from various environments including both aquatic and terrestrial, and often from plant materials [15–22]. However, many strains of these, as well as other relevant species, have not yet been studied in detail for properties relevant for oil production. An efficient strain is key in successful bio-production, which motivates further screening and selective isolation from nature.

Oleaginous yeasts typically accumulate oil in the form of triacylglycerols (TAG) under limiting conditions of a nutrient other than carbon [2, 8, 11, 23, 24]. Commonly, nitrogen limitation is used to initiate lipid production [11, 25]. The carbon-to-nitrogen ratio (C/N ratio), temperature, pH and overall nutrient composition affect the biomass and lipid yield [8, 11, 14, 25–27]. In addition, the extraction method for FA analysis may affect the apparent FA composition [2, 8, 28, 29]. The FA composition is nutritionally important in food and feed applications, and functionally for biodiesel [3, 30].

Few studies address lipid accumulation in yeast utilizing xylose as the sole carbon source, or yeast grown in lignocellulosic hydrolysate. Research into lipid accumulation under these conditions is important for achieving high yields of lipids, as well as improving the economic viability of sustainable plant waste hydrolysates as yeast growth medium. With this study, we address this issue by investigating lipid yields on a wide variety of substrates, including lignocellulosic hydrolysate.

In the present work we describe (i) a strategy for selective isolation of oleaginous yeasts from nature, (ii) screening of growth on different substrates such as xylose and glycerol, (iii) screening of lipid production capacity and FA composition using different substrates including plant hydrolysates, (iv) identification and (v) detailed production studies of selected high-lipid production strains. The best performing yeasts found in this work are promising organisms for further investigation into future sustainable applications of in food, feed and fuel.

## Results

### Yeast isolation, identification, and screening for oleaginicity

Most yeasts were isolated from a variety of plants in the greenhouses of the Botanical Garden and Trädgårdsföreningen, both in Gothenburg, Sweden. As a contrast to the warm humid greenhouses, yeasts were also isolated from plant material collected in the mountains of Gran Canaria, and on the island Brännö in the Southern Gothenburg archipelago, with the latter being a much windier and colder environment. To select for pentose utilizing strains (essential for high yield from lignocellulose substrates), xylose was consistently used as the sole carbon source in the first isolation medium. Chloramphenicol was used to supress bacterial growth. This resulted in 30 different strains, which were then screened for their oleaginous properties.

Our screening data for the isolated strains in lipid inducing medium showed a correlation between habitat in which the yeast was isolated and fatty acid content. Yeasts isolated from the greenhouses generally showed higher FA content than yeasts isolated from more windy and cold areas (Table 1). Yeast strains from the greenhouses of the Botanical Garden generally showed the highest presence of oleaginous strains, 11 out of 13 strains from the initial screening had a fatty acid above 20%. In contrast, the four analysed yeasts isolated on the Swedish island Brännö (most of the year far colder than the greenhouses, including icy winters) showed only 5.3, 6.3, 8 and 13.6% from the initial screening. This suggests that the warm and humid greenhouse conditions favour growth of oleaginous yeasts.

**Table 1:** Yeast annotation and the location of origin. Yeast species (where applicable) and fatty acid content from initial fatty acid accumulation screening on glucose based medium in % per cell dry weight (DW). Species marked n.i indicates species not identified

Yeast isolate (place and annotation)	Species	FA content initial screen (% of DW)
*Palmhuset. Gothenburg*		
PAL-Fa	n.i	34.3
PAL-Fb	n.i	16.3
PAL-Fd	n.i	19.1
PAL-Ia	n.i	7.2
PAL-Ib	n.i	18.2
PAL-Ja	n.i	13.3
PAL-Ha	n.i	14.5
PAL-Pa	n.i	18.2
PAL-Pb	n.i	12.3
PAL-C	n.i	12.0
*Botanical Garden. Gothenburg*		
BOT-10.2	n.i	15.0
BOT-10.3	*Sporidiobolus salmonicolor*	26.8
BOT-O	*Pseudozyma hubeiensis*	46.8
BOT-A1	n.i	21.3
BOT-A2	*Rhodosporidium toruloides*	40.4
BOT-I	n.i	23.7
BOT-1	*Rhodotorula minuta*	34.0
BOT-6.1	*Cryptococcus flavescens*	41.4
BOT-6.2	*Rhodotorula minuta*	34.4
BOT-J.1	*Rhodotorula* sp.	49.0
BOT-J.2	n.i	17.0
BOT-4	*Rhodotorula glutinis / Rhodosporidium diobovatum*	32.5
BOT-8	*Rhodosporidium toruloides*	46.8
*Gran Canaria. Spain*		
GC-7	n.i	18.5
GC-9	n.i	19.3
GC-12	n.i	12.6
*Brännö. Gothenburg*		
BR-c.a	n.i	13.6
BR-g.a	n.i	5.3
BR-h.a	n.i	8.0
BR-h.ar	n.i	6.3
*Reference strains*		
CBS14	*Rhodosporidium toruloides*	29.1
CCUG32821	*Rhodotorula glutinis*	35.0

Three strains from the Botanical Garden reached between 46.8 and 49% FA content in the screening. However, in addition to high specific lipid content, the achievable cell density from a given amount of substrate is also crucial for a production strain. The strains with the strongest combination of high cell density after cultivation, and high FA content per yeast biomass, were *Pseudozyma hubeiensis* BOT-O (46.8% FA and OD_600nm_ 32), *Rhodosporidium toruloides* BOT-A2 (40.4% FA and OD_600nm_ 29), *Cryptococcus flavescens* BOT-6.1 (41.4% FA and OD_600nm_ 30), and *Rhodosporidium toruloides* BOT-8 (46.8% FA and OD_600nm_ 33).

We also observed that yeast isolation on a xylose-based medium gave a relatively high amount of pigmented oleaginous yeasts. The genera *Rhodotorula* and *Rhodosporidium* clearly dominated among the identified isolates in this study, representing 7 out of 9 identified strains (Table 1). Strains for identification were selected according to their FA profiles (composition and amount of FA) in the screening (Supplementary Figure S1), as well as being microscopically, macroscopically and phenotypically different. The accession numbers for the identified species are found in Supplementary Table S2. Identified strains will be referred to by species and strain name in all experiments.

### Growth performance on different solid media

The growth screenings on agar-based media revealed that no strains were able to grow on the Dissolving Lut (DL) medium at the concentration range 50–100%, and only two strains, PAL-Pb and *Sporidiobolus salmonicolor* BOT-10.3, were able to grow on the 25% concentration of this medium (Table 2). *S. salmonicolor* 10.3 was also the only strain able to grow in the full concentration of the Enzymatic Wheat Hydrolysate (EWH) medium, and showed very strong growth on 50% EWH medium. All tested strains were able to grow on the 25% concentration of the EWH medium as well as on all Yeast Nitrogen Base (YNB) based media containing either glucose, xylose or glycerol. The growth capacity for selected strains were estimated by visual inspection of colony forming capacity (Table 2).

**Table 2:** The results from growth performance for each strain on different media and carbon sources is given as strong growth (+++), slightly impaired growth (++), strongly impaired growth (+) or no growth (−). EWH is enzymatically treated wheat hydrolysate, and DL is dissolving Lut. The percentages indicate to which level the original substrate was diluted to, with milliQ water

Strain	Glucose	Xylose	Glycerol	EWH 25%	EWH 50%	EWH 75%	EWH 100%	DL 25%	DL 50%	DL 75%	DL 100%
PAL-Fa	++	+	+	++	+	–	–	–	–	–	–
PAL-Fb	++	+	+	+	+	–	–	–	–	–	–
PAL-Fd	++	+	+	+	+	–	–	–	–	–	–
PAL-Ia	++	+	+	+	–	–	–	–	–	–	–
PAL-Ib	+	++	+++	++	+	–	–	–	–	–	–
PAL-Ja	++	+	++	+	–	–	–	–	–	–	–
PAL-Pa	+	++	+++	++	+	–	–	–	–	–	–
PAL-Pb	+	+	++	+	+	+	–	+	–	–	–
BR-ca	++	+	++	++	+	–	–	–	–	–	–
GC-7	+++	+	+++	+++	++	+	–	–	–	–	–
GC-9	+++	+	++	+++	++	+	–	–	–	–	–
BOT-10.3	+	+	++	+++	+++	+	+	+	–	–	–
BOT-O	n/a	n/a	n/a	n/a	n/a	n/a	n/a	n/a	n/a	n/a	n/a
BOT-A1	++	+	++	+++	++	+	–	–	–	–	–
BOT-A2	++	+++	++	++	+	–	–	–	–	–	–
BOT-1	+++	+++	++	++	+	–	–	–	–	–	–
BOT-6.1	++	+++	+	++	+	–	–	–	–	–	–
BOT-6.2	+++	++	+++	++	+	–	–	–	–	–	–
BOT-J.1	++	++	+	++	+	–	–	–	–	–	–
BOT-4	+	++	++	++	+	–	–	–	–	–	–
BOT-8	+++	+++	+	++	+	–	–	–	–	–	–
CCUG 32821	+++	+	++	++	+	–	–	–	–	–	–
CBS 14	+++	–	+	+	+	–	–	–	–	–	–

^a^ BOT-O showed poor overall growth on solid media, hence the screening for this particular isolate was performed solely in liquid media

*Pseudozyma hubeiensis* BOT-O showed generally poor growth on solid media, and in a few cases the colonies from this strain grew taller, rather than growing wider, as is usually the case on solid media. However, this strain showed strong growth in the liquid versions of the media (described later).

With respect to growth on xylose-based solid medium, four of the Botanical Garden strains, *Rhodosporidium toruloides* BOT-A2, *Rhodotorula minuta* BOT-1, *Cryptococcus flavescens* BOT-6.1 and *Rhodosporidium toruloides* BOT-8, showed very strong growth, similar or stronger than on the glucose based medium.

### Lipid accumulation screening

Based on the growth performance on the different solid media, a subset of strains was selected for initial screening of lipid accumulation in the corresponding liquid media. The results of the screening, total FA content, and strain specific FA profiles are presented in Supplementary Figure S1. The detailed data is also presented in Supplementary Table S1.

The highest total FA levels per biomass unit obtained in the screening among the isolated yeasts were found in strain *P. hubeiensis* BOT-O (46.8% in glucose, 43.2% in xylose and 38.9% in glycerol), *R. toruloides* BOT-A2 (40.4% in glucose, 27.3% in xylose, 42.1% in glycerol and 45.1% in EWH) and *R. toruloides* BOT-8 (46.8% in glucose, 19.7% in xylose, 36.8% in glycerol and 51.8% in EWH). Strain *P. hubeiensis* BOT-O accumulated 43.2% FA on the xylose based medium, which corresponds to 58% more FA than the second-best strains BOT-A1 (not identified) and *R. toruloides* BOT-A2 (27.3%) and reference strain *Rhodotorula glutinis* CCUG32821 (27.4%).

The FA profiles of a single strain varied depending on type of substrate used, as seen in Supplementary Figure S1 and Supplementary Table S1. In strain *P. hubeiensis* BOT-O for example, no FA longer than C18 (C18:1 and C18:2) were found in the glucose based medium, whereas in both the glycerol based medium and the xylose based medium, C20:0 and C22:0 were detected. Additionally, in the xylose based medium C24:0 was detected as well. In strain *R. toruloides* BOT-A2, we were also able to detect C18:3 in all tested media, reaching 7.2 mg/g dry weight of yeast in the EWH medium. The highest level of C18:3 among the isolated strains was found in strain *Sporidiobolus salmonicolor* BOT-10.3, cultured in the glucose medium. In this strain the content of linolenic acid (C18:3) was found to be 9.2 mg/g dry weight yeast biomass.

**Fig. 1:**
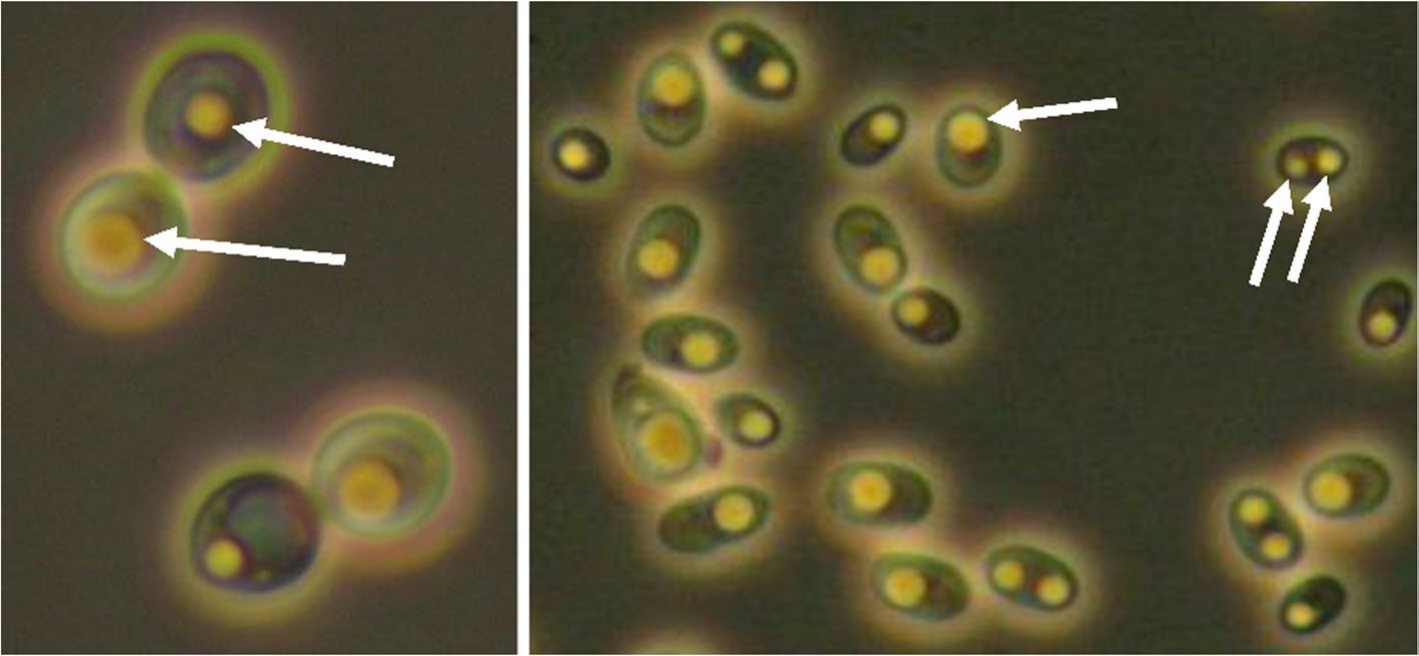
Phase contrast microscopy photos of *Rhodosporidium toruloides* BOT-A2 (left photo) and *Pseudozyma hubeinensis* BOT-O (right photo). Storage lipids are seen as bright/yellow lipid bodies inside the cells, indicated by arrows

The lipid accumulation was also monitored using a phase contrast microscope, with which the lipid droplets were easily detectable inside the yeast cells (Fig. 1). It was found that within a genetically identical yeast population, there were some variations in the number and size of lipid droplets (assessed by microscopy) between individual cells, as well as a temporal variance in lipid droplet morphology. At later stages of cultivation, it was more common to observe fewer but larger droplets covering approximately 2/3 of the cell section area. At earlier stages of cultivation, multiple small droplets generally dominated over large droplets. It appears that as the lipid content increases, smaller droplets merge to form larger ones.

### Fermenter studies of strain BOT-O, BOT-A2 and CCUG32821

**Fig. 2:**
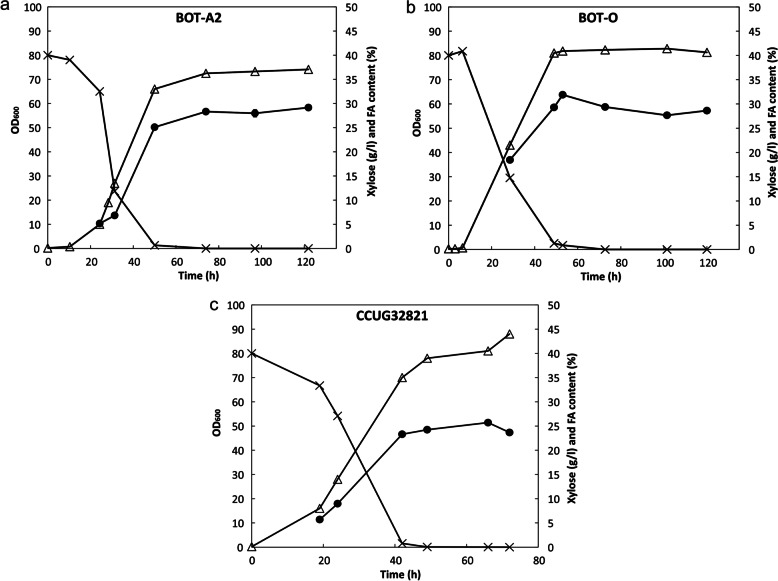
The growth as OD_600 nm_ (Δ), the xylose concentration (×) and the fatty acid accumulation (●) in cells (on a dry weight biomass basis) was monitored during cultivations with **a** strain *Rhodosporidium toruloides* BOT-A2, **b** *Pseudozyma hubeinensis* BOT-O and **c** *Rhodotorula glutinis* CCUG32821. The data points are mean of duplicates, with standard deviations less than 4% in all cases

Three strains were selected from the screenings to be further studied for lipid accumulation in xylose-based fermenters. The growth (optical density at 600 nm), the xylose consumption and the FA accumulation (% per dry weight biomass) during cultivations are presented in Fig. 2. The results show that all strains had consumed all xylose in the culture media within approximately 50 h. The reference strain, *R. glutinis* CCUG32821, had a longer lag phase compared to the three other strains. The cultivation of strains *P. hubeiensis* BOT-O and *R. toruloides* BOT-A2 was monitored over the course of 120 h, while *R. glutinis* CCUG32821 was monitored for 70 h. Both strain *P. hubeiensis* BOT-O and *R. toruloides* BOT-A2 show a rapid increase in FA content over the first 50-60 h, thereafter a stagnation in the FA accumulation. The FA level in these experiments reached approximately 32% (dry weight biomass basis) for *P. hubeiensis* BOT-O, 28% for *R. toruloides* BOT-A2, and 24% for *R. glutinis* CCUG32821.

### Comparative shake flask studies of strain BOT-O and BOT-A2 on glucose and xylose

**Fig. 3:**
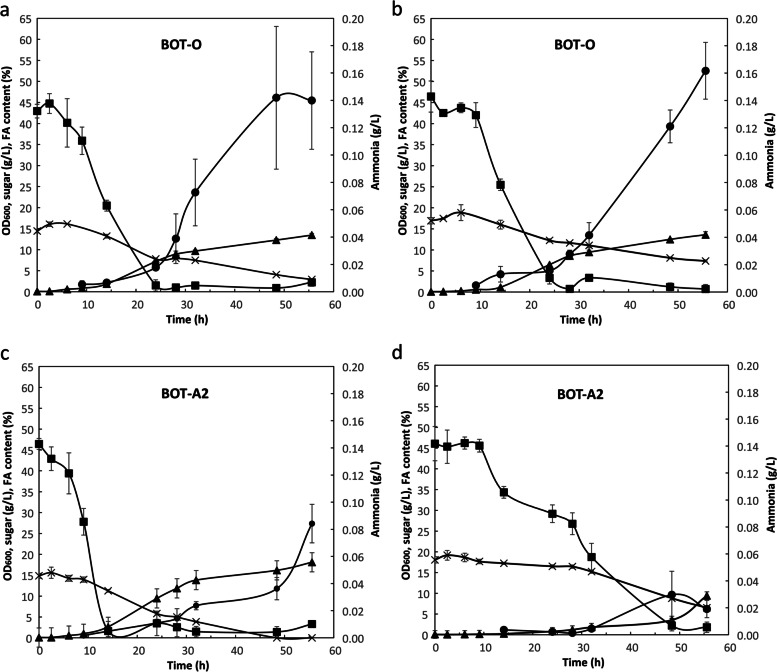
Comparative shake flask cultivations for *Pseudozyma hubeinensis* BOT-O and *Rhodosporidium toruloides* BOT-A2 with glucose or xylose as carbon source. **a** BOT-O on glucose, **b** BOT-O on xylose, **c** BOT-A2 on glucose, **d** BOT-A2 on xylose, with OD_600nm_ (▲), sugar concentration (×), fatty acid accumulation (●) and ammonia concentration (◼). All conditions were performed in triplicates

Two strains, *P. hubeiensis* BOT-O and *R. toruloides* BOT-A2, were selected to study the effects of different carbon sources on growth, carbon and nitrogen utilisation, and lipid accumulation. The C/N ratio of 80 was chosen to acquire a good overview over the non-limiting exponential growth phase, and also have sufficient remaining sugar in the nitrogen limitation phase. Results for *P. hubeiensis* BOT-O are presented in Fig. 3a and b and for *R. toruloides* BOT-A2 in Fig. 3c and d, with lipid and biomass yields presented in Table 3.

**Table 3:** Fatty acid and biomass yields and rates, calculated during nitrogen starvation from shake flask cultivation on single sugars glucose and xylose. This includes timepoints between 24 h – 56 h for BOT-O on glucose and xylose, and BOT-A2 on glucose, as well as 32–56 h for BOT-A2 on xylose. For better comparisons yields were also calculated as an hourly rate

Strain	Sugar	Y_P/S_ (g/g)	Hourly Y_P/S_ (g/g*h)	Y_X/S_ (g/g)	Hourly Y_X/S_ (g/g*h)
BOT-O	glucose	0.06 ± 0.03	1.9 × 10^−3^ ± 1.0 × 10^−3^	0.15 ± 0.04	2.7 × 10^−3^ ± 1.2 × 10^−3^
BOT-O	xylose	0.07 ± 0.03	2.2 × 10^−3^ ± 0.9 × 10^−3^	0.15 ± 0.04	2.7 × 10^− 3^ ± 1.2 × 10^− 3^
BOT-A2	glucose	0.06 ± 0.01	1.5 × 10^− 3^ ± 0.4 × 10^− 3^	0.25 ± 0.01	4.5 × 10^− 3^ ± 0.5 × 10^− 3^
BOT-A2	xylose	0.01 ± 0.00	0.2 × 10^− 3^ ± 0.1 × 10^− 3^	0.15 ± 0.03	2.6 × 10^− 3^ ± 0.9 × 10^− 3^

For *P. hubeiensis* BOT-O, growth on xylose and glucose resulted in similar OD_600nm_, ammonia uptake and lipid and biomass production. Growth rates were similar, with a μ_max_ of 0.18 ± 0.01 h^− 1^ and 0.19 ± 0.00 h^− 1^ for growth on glucose and xylose respectively. Nitrogen was fully depleted in both substrates after 24 h, and after 56 h the yeast had used most of the glucose (20.0 ± 0.8% remaining), while the xylose concentration (7.4 ± 0.2 g/L, 43.5 ± 0.2% remaining) was still high in the medium (Fig. 3a and b). Lipid production increased after nitrogen depletion and reached 45.5 ± 11.6% and 52.6 ± 6.7% FA per cell dry weight after 56 h for growth on glucose and xylose, respectively. Overall, lipid yields and lipid production rates were slightly higher for strains grown on xylose (Table 3) during nitrogen starvation. The biomass yield was similar during the nitrogen starvation phase, with 0.15 ± 0.04 g/g for both in the same time frame (Table 3).

In contrast to BOT-O, growth on glucose and xylose generated different results for *R. toruloides* BOT-A2. Results for *R. toruloides* BOT-A2 on glucose were similar to those of BOT-O, but BOT-A2 growth on xylose differed significantly. The exponential growth phase was much longer on xylose (48 h vs. 24 h on glucose), with maximum OD_600nm_ of 18.1 ± 1.2 on glucose and 9.4 ± 0.7 on xylose (Fig. 3c and d). The growth rate μ_max_ for growth on glucose was more than double that on xylose with 0.20 ± 0.01 h^− 1^ compared to 0.09 ± 0.00 h^− 1^ respectively. Similar results have been reported for the *R. toruloides* NP11 strain [31]. Glucose was fully depleted after 48 h, whereas 36.0 ± 1.0% xylose remained in the xylose cultivation after 56 h. Nitrogen was depleted after 32 h in the glucose cultivation and after 48 h in the xylose cultivation.

Lipid production increased after nitrogen depletion in growth on glucose. The highest lipid content was measured after 56 h with 27.4 ± 4.6% on glucose and only 5.1 ± 1.0% on xylose. In contrast to BOT-O, lipid yields and production rates for BOT-A2 varied substantially between glucose and xylose (Table 3), with the lipid production rate during nitrogen starvation being 7-fold higher on glucose than on xylose. However, biomass yields were significantly lower on xylose (Table 3), which impacted the overall lipid yield. Longer cultivation times for A2 on xylose might be beneficial for lipid production, as was done in the fermenter experiment where BOT-A2 on xylose produced a significantly higher amount of lipids.

Comparison between BOT-O and BOT-A2 in terms of lipid and biomass yields also shows that, while biomass yields and biomass production rates during nitrogen starvation are similar for BOT-O on glucose and xylose as compared to BOT-A2 on xylose, BOT-A2 on glucose showed a significant difference, with the biomass yield and hourly biomass production rate being approximately 1.6-fold higher for BOT-A2 on glucose (Table 3). Overall, lipid production yields and rates were comparable for BOT-O on both sugars and BOT-A2 on glucose, but significantly higher than those for BOT-A2 on xylose (Table 3).

## Discussion

This work presents isolation of various yeasts from nature which demonstrated natural ability to utilize xylose, a high capacity to accumulate lipids, and also the ability to produce pigments such as carotenoids in some cases. The initial selection, favouring the ability to grow on xylose as the sole carbon source, resulted in a high number of oleaginous yeasts. A total of 15 out of 36 pigmented isolates showed a fatty acid content above 18%, which would be higher if glycerol (as part of triacylglycerols) was included in the weight, and if culture conditions were optimized. According to literature, about 5% of all yeast species are able to accumulate lipids above 25% [8]. In our study, however, 28% of the pigmented isolates had a fatty acid content above 25%, selection for xylose growth and pigmentation in our case seemed to have favoured oleaginous yeasts. Our results also suggest a correlation between oleaginicity and warm, humid and wind-free environments (such as the greenhouse of the Botanical Garden). The high level of oleaginous yeasts among the pigmented isolates may suggest a protective role of carotenoids against lipid oxidation in lipid accumulating yeasts, and perhaps that yeasts living in areas with high ultraviolet (UV) exposure require pigmentation as protection against the UV radiation.

The strains selected for sequencing were identified as *Sporidiobolus salmonicolor* (BOT-10.3), *Pseudozyma hubeiensis* (BOT-O), *Rhodosporidium toruloides* (BOT-A2, BOT-8), *Rhodotorula minuta* (BOT-1, BOT-6.2), *Cryptococcus flavescens* (BOT-6.1), *Rhodotorula oligophage (BOT-J.1)* and *Rhodotorula glutinis* (BOT-4). These showed strong lipid accumulation in at least some media conditions, with several showing strong lipid accumulation in all media conditions tested. In this study, *P. hubeiensis* BOT-O and *R. toruloides* BOT-A2 for instance reached 38.9–46.8% and 27.3–45.1% of FA content per cell dry weight respectively, with variations in production seeming to be most influenced by the growth medium. These two strains show a high capacity for converting the pentose xylose to oil (above 40% and near 30%, respectively), which is beneficial for the efficient utilisation of lignocellulose-based substrates such as forestry and agricultural wastes. *P. hubeiensis* BOT-O was inhibited when cultured on agar-based media, however showed growth comparable to the other isolated yeast strains in the liquid version of the same media. The reason for this discrepancy is not known. The knowledge about this species is still limited, and more research is required. However, we could observe that growth of BOT-O on xylose and glucose did not show a significant difference in terms of biomass accumulation, growth rate and lipid accumulation, demonstrating that this strain, in contrast to other yeasts, catabolizes pentose sugars as efficiently as it does hexose sugars. This was also reported by Tanimura et al. [32] and makes *P. hubeiensis* BOT-O a very interesting yeast for utilisation of renewable feedstocks containing pentose sugars, such as lignocellulosic hydrolysates. This species has previously been shown to be a producer of biosurfactants (glycolipids) [33], but we show here that they are also producers of storage lipids under nitrogen starvation on different carbon sources.

Previous studies and reviews on lipid accumulation by some of the same species found in this work show a large variation in lipid content [34–36]. One publication presents between 10 to 38% lipids for 6 strains of *P. hubeiensis* Y277 [35], and the work by Wei et al [36] reports 18.6% FA in *R. toruloides,* and 20.3% in *Rhodotorula graminis*. Comparing this with our data suggests a high inherent lipid accumulating capacity in our strains, which can likely be further optimized by changing parameters such as pH, temperature, dissolved oxygen level and nutrient composition, as well as C/N ratio and carbon concentration. In previous studies on pigmented yeasts a lipid content of 52.5% per cell dry weight for *R. glutinis* CGMCC 2.703 was achieved after process optimization by using a two-stage pH regulation, a 60% lipid content yield was obtained from *R. glutinis* BCRC22360 by optimization of the dissolved oxygen level [37], 61% lipid content from *R. glutinis* TISTR5159 was achieved in an optimized bioreactor [38], and a lipid yield of up to 76% in an engineered *R. toruloides* IFO0880 strain [39]. This suggests a strong potential to improve the lipid accumulation of our strains through various methods.

The amount of FA produced by *P. hubeiensis* BOT-O in our fermenter studies was lower (about 30% FA per dry weight biomass) than in the shake flasks (about 43–53%, depending on the carbon source). Our fermenter conditions appeared not as favourable for lipid accumulation in *P. hubeiensis* as the shake flask conditions used in this study, but could be optimized. Even in the shake flask experiment with a C/N ratio of 80 more lipids were produced than in the fermenters, even though this C/N ratio is less favourable for high specific lipid content [25].

For strains *R. toruloides* BOT-A2 and *R. glutinis* CCUG32821 on the other hand, the FA levels were maintained at approximately the same levels in the fermenter studies as in the initial shake flasks. During the shake flask experiment with C/*N* = 80 on xylose however, the lipid content was significantly lower, at only 5.1%. This could be the result of the less beneficial C/N ratio and slower initial growth, which resulted in less biomass, as well as the short cultivation time (56 h compared to 120 h in the fermenter studies). Indeed, in literature it has been shown that lipid content for *R. toruloides* can be comparable between cultivation on glucose and xylose, but requires a significantly longer cultivation time on xylose [40].

In the C/N = 80 shake flask cultivations a difference between BOT-O and BOT-A2 was detected regarding lipid and biomass production yields and rates: BOT-O had slightly higher lipid production rates on both sugars with 1.9 × 10^− 3^ ± 1.0 × 10^− 3^ g/g*h on glucose and 2.2 × 10^− 3^ ± 0.9 × 10^− 3^ g/g*h on xylose during nitrogen starvation compared to BOT-A2 on glucose with 1.5 × 10^− 3^ ± 0.4 × 10^− 3^ g/g*h during nitrogen starvation (Table 3). This might be related to the biomass production yields and rates being higher for BOT-A2 grown on glucose during nitrogen starvation (Table 3) compared to BOT-O on both sugars. BOT-A2 metabolism during nitrogen starvation appears to prioritize growth more than metabolism in BOT-O, potentially even using storage lipids and thus reducing the overall lipid yield. Overall, lipid yields were lower in our C/*N* = 80 shake flask experiment for *R. toruloides* BOT-A2 compared to another *R. toruloides* strain IFO0880: Zhang and colleagues achieved yields of 0.09–0.12 g/g for cultivations on glucose compared to our 0.06 g/g, and 0.10 g/g on xylose (compared to our 0.01 g/g) [39, 40]. Among other things, however, cultivation times were significantly different with between 144 and 217 h for glucose cultivations and 385 h for the xylose cultivation in their work. Engineered strains have been shown to be able to produce nearly double the lipid yield in a similar time frame [39, 40], making metabolic engineering an interesting strategy for increasing lipid production in *R. toruloides* BOT-A2.

In the fermenter studies, *R. glutinis* CCUG32821 appeared to have a second phase of growth, starting at 68 h. This could be a result of the strain utilizing released lipids from old lysing cells for its growth, which is supported by the observed decrease in specific FA content from this time-point.

The lignocellulose substrate EWH was shown to yield high biomass-specific lipid accumulation in some strains. However, this coincided with low biomass production. This may be either due to limitations in specific nutrients or restricted growth by inhibitors commonly present lignocellulose substrates, such as furfural and hydroxymethylfurfural. Both of these potential issues can be addressed and improved in future studies. *R. toruloides* strains have been shown to have a tolerance towards other lignocellulosic inhibitors such as acetic acid, whereas other oleaginous yeast strains were also able to grow well in the presence of furfural [41]. Evolutionary or genetic engineering can be applied to improve the ability of strains to grow in the EWH medium, by further improving tolerance towards inhibiting compounds [42]. No engineering, however, is necessary to achieve efficient pentose utilization, which makes these isolated oleaginous yeast strains better production hosts than model species such as *S. cerevisiae* and *Y. lypolytica*, naturally unable to utilize pentoses.

Depending on the intended use of the microbial lipids, different FA compositions are desired. To produce biodiesel with good cold flow properties, *R. toruloides* BOT-A2 may be explored as it showed a high level of unsaturated FA (61–67% in all substrates tested). To produce an oil with a high level of long chain (LC) FA (> C18:n) *P. hubeiensis* BOT-O may be investigated in a xylose based medium, as it resulted in 6.4% of LCFA, or strain *R. minuta* BOT-6.2 in EWH medium which gave 6.7% LCFA. For an oil with PUFA, several of the strains show high percentage of PUFA’s, but for an oil containing the FA 18:3, strains *S. salmonicolor* BOT-10.3, BOT-A1, *R. toruloides* BOT-A2, BOT-I, *R. glutinis* BOT-4 and *R. toruloides* BOT-8 produce FA 18:3 (over 1.4%) as well as a total FA level above 20% in one or more of the tested media.

Lipid production using oleaginous yeast has several benefits over other production organisms. For example, algal lipid production reaches similar FA content per on a dry weight basis, but with much lower total biomass yield than yeast. *S. cerevisiae* strains need to be genetically engineered to produce FA, while oleaginous yeasts do so naturally. Plant lipid production means longer cultivation times and growth cycles as well as a dependence of climate and season which can be avoided using yeasts growing in bioreactors. Plant lipid production for fuels is under constant debate concerning i) the competition with food production, ii) unsustainable and harmful use of pesticides and fertilizers that harms land, animals and workers, and iii) the deforestation and its devastating effects. Those negative impacts could be reduced, or even eliminated, using yeast for lipid production.

Climate change, growing human population, increased need of plant oil croplands and the competition between fuel and food production highly motivates research and development of alternatives to traditional plant oil biodiesel and fossil fuel production. The isolated oleaginous yeast strains in this work naturally utilize xylose and glycerol in addition to glucose, and were found to accumulate high levels of storage lipids with a FA composition similar to certain plant oils. Our work demonstrates an efficient way to isolate yeasts with suitable properties, and showcases strains with a high potential for further development for biobased production of oils for applications in food, feed and fuel.

## Conclusions

In this study, we have found various natural isolates with high lipid production capabilities and ability to grow not only on glucose, but also xylose, glycerol and natural plant hydrolysate. *R. toruloides* BOT-A2 and *P. hubeiensis* BOT-O specifically show great potential for becoming highly interesting production strains, with high levels of storage lipids compared to currently used production strains and good growth on various substrates. In BOT-O glucose repression was not detectable, making it particularly desirable for utilization of plant waste hydrolysates. Furthermore, the isolated strains were shown to produce oils with fatty acid profiles similar to that of plant oils, making them interesting for future applications in fuel, food or feed production.

## Materials and methods

### Media and chemicals

The isolation medium was designed to select for xylose utilizing yeasts and consisted of yeast extract (10 g/L, Scharlau), peptone (20 g/L, Bacto, BD), xylose (10 g/L, Serva) and chloramphenicol (0.2 g/L, Biochemika). The medium was autoclaved, followed by addition of filter sterilized (0.22 μm pore size) chloramphenicol (Sigma Aldrich). For solid medium, agar (20 g/L, Bacteriological, Scharlau) was added.

Yeast growth performance was assessed on agar based and liquid versions of five different media. Three defined media were prepared from yeast nitrogen base (YNB) without ammonium sulphate (Difco) in succinate buffer (pH 5.5), with addition of nitrogen at 5 g/L ammonium sulphate (Scharlau), and addition of either glucose, xylose or glycerol to a C/N molar ratio of 10. Additionally, two natural hydrolysates were used; one dissolving lut (DL) from Domsjö processes, pH adjusted to pH 5.5 with 5 M NaOH (Sigma), and one EWH, filtered and pH adjusted to pH 5.5 with 1 M NaOH (from the group Industrial Biotechnology, Chalmers University of Technology). The two natural substrates were tested at four different concentrations, with 100, 75, 50% or 25% of original solution, diluted with sterile milliQ water.

Lipid accumulation of selected yeasts was determined after cultivation in seven different media. Firstly, three YNB based media, in succinate buffer (pH 5.5), supplemented with either glucose, glycerol or xylose, plus ammonium sulphate to yield a C/N ratio of 110 for glucose and glycerol media (20 g/L), and 55 for xylose medium (10 g/L). Secondly, four natural substrates were used; one DL and one EWH, both diluted to 25% with milliQ, and two differently prepared molasses with addition of sugar (2% w/v), methionine, leucine and histidine. Phosphate was then added to one of the two media preparations, which were then referred to as Molasses+P or Molasses-P (without addition of phosphate).

YPD medium (yeast extract 10 g/L, peptone 20 g/L, glucose 20 g/L,) was used for short term storage. For solid medium agar (20 g/L) was added. Long term storage was done in 15% glycerol (Sigma) solution in cryotubes at − 80 °C.

### Collection of plant material and isolation of yeast

Plant material for yeast isolation was collected at four different occasions and locations: Palmhuset in the Garden Society of Gothenburg (Trädgårdsföreningen), Brännö outside of Gothenburg, the greenhouse of the Botanical Garden in Gothenburg, and from the mountains on Gran Canaria, Spain. Leaves and twigs were collected using surface sterilized scissors and tweezers, and deposited in clean plastic bags for storage and transportation of samples. All collected samples were used for isolation within 3 days of collection. Permission for collecting plant material was obtained from the Garden Society of Gothenburg (Trädgårdsföreningen) and the Botanical Garden in Gothenburg. This study complies with all the relevant institutional, national and international guidelines and legislations for plant ethics.

Yeast isolation on solid media was done either by directly placing the plant sample on the agar surface, or by repeatedly pressing the plant sample onto the agar surface to allow microorganisms to be transferred onto the agar. After incubation, pigmented yeast colonies were picked and cultivated until pure, by repeat plate spreading on xylose based medium.

Some plant samples resulted in strong growth of mould, which made it difficult to isolate yeast colonies from the agar-based growth media. To overcome this, a simple separation method was implemented. The plant sample was first placed in 5 ml of isolation medium in a 15 mL Falcon tube. After sufficient growth had occurred (usually 24–48 h) the plant sample was aseptically removed from the tube, and the tube was left standing upright for 2–3 hours, to allow for the yeast cells to sediment. Since yeasts sediment faster than moulds, a sample with a higher yeast/mould ratio was subsequently withdrawn from the bottom of the tube. If needed, this procedure was repeated, before continuing the isolation on solid medium.

### Oleaginous reference strains

The strain *Rhodosporidium toruloides* CBS14 was purchased from Centraalbureau voor Schimmelcultures (CBS), and the strain *Rhodotorula glutinis* CCUG32821 from Culture Collection, University of Gothenburg (CCUG).

### Growth and lipid accumulation screenings

An initial screening for fatty acid accumulation on a glucose-based medium was performed in order to limit the number of isolates to be included in this study. The growth performance of each isolate was first assessed on different solid media; glucose based, xylose based, glycerol based and various concentrations of DL and EWH media. The degree of growth was examined by serially diluting samples of each strain, starting from a known OD, and placing 10 μl drops from the set of dilutions onto agar-based media. The growth of the drop-series on the plates were visually estimated by studying i) colony number at the various dilutions, ii) colony sizes, iii) colony morphology (shape, colour development, formation of hyphae) and iii) colony formation time. The growth was judged as strong (+++), mildly inhibited (++), inhibited (+) or not growing (−). The growth was benchmarked against growth on glucose.

For liquid culture screenings, single cultures without replicates were performed, and the final biomass was analysed for its fatty acid content as a first screening. Precultures were done in 4 mL of YPD medium in 15 mL Falcon tubes overnight at 30 °C. The oil production screening was performed in shake-flask batch cultures containing 25 mL YNB with glucose as the carbon source and a C/N ratio of 110. The starting OD_600nm_ was 0.5, and the flasks were incubated in Barnstead Lab-line Max^Q^ 4000 (Skafte Medlab) incubator at 30 °C and 180 rpm. Only strains with adequate growth were used for lipid accumulation analysis.

Optical density was assessed using an Ultrospec 10 spectrophotometer (Amersham, Biosciences). Formation of lipid bodies inside the yeast cells was visually observed using a ZEISS HBO 50_/AC_ Axiostar plus microscope. A fixed cultivation time of 95 h was chosen for the screenings, based on i) observation of lipid accumulation through microscopic pre-studies and ii) support by previous literature [8].

After incubations, cells were harvested by centrifugation and the cell pellets were washed once in sterile milliQ water, subsequently lyophilized, and stored at − 20 °C for maximum 1 week before lipid extraction and quantification. Strains with a fatty acid content above 18% on a dry weight basis are annotated as oleaginous in this work.

### Fatty acid analysis

The FA content was analysed using a modified version of the KOH/ethanol extraction method described by Andlid et al. [29] A volume of 250 μl internal standard of fatty acid 17:0 (4 mg/mL) in toluene was added to 25 mg of dry biomass sample, followed by addition of 5 mL of 2.14 M KOH (in 12% EtOH) and incubation for 2 h at 70 °C. After incubation, samples were acidified to pH 2 by addition of 2.5 mL of 5 M HCl, followed by extraction of FAs with 4 + 3 + 3 mL hexane. Extracts were pooled before evaporation under a flow of nitrogen gas at 40 °C. FAs were methylated by addition of 1 mL of 10% acetyl chloride in methanol and 1 mL toluene, followed by incubation for 2 h at 70 °C. The incubation was followed by addition of 0.4 mL milliQ and 2 mL petroleum ether / diethyl ether (80:20). After thorough mixing, the upper phase was collected and evaporated under a flow of nitrogen gas at 40 °C. The samples were then suspended in 2 mL isooctane before analysis and storage. For analysis, 0.5 mL sample was diluted with 0.5 mL isooctane into GC-MS vials.

The fatty acid methyl esters (FAME) were analysed using Agilent technologies GC-MS (USA: GC 7890A, MSD 5975C). The column was a DB-WAX with properties 0.25 × 30 mm and 0.25 μm film thickness. A constant flow of 1 mL/min was used, with helium as the carrier gas. The split detector was set to 275 °C with a split ratio of 15:1. The column was temperature programmed to start at 100 °C followed by an increase of 4 °C/min until 250 °C was reached and held for 4 min before decreasing back to 100 °C. The mass selective detector (MSD) was calibrated with MIX100 from Larodan (Malmö, Sweden). The FA content of the analysed sample was calculated using the internal standard.

### Identification of yeast isolates

Genetic identification was done for a small sub-set of strains, including *S. salmonicolor* BOT-10.3, *P. hubeiensis* BOT-O, *R. toruloides BOT*-A2, *R. minuta* BOT-1, *C. flavescens* BOT-6.1, *R. minuta* BOT-6.2, *R. oligophage* BOT-J.1, *R. glutinis/R. diobovatum* BOT-4 and *R. toruloides* BOT-8. The strains were selected based on their presentation of interesting lipid production profiles, as well as appearing microscopically, macroscopically and phenotypically different. The identification was performed using the rRNA method according to Kurtzman and Robnett (1998) [43]. Biomass of 10 mL YPD culture was collected and resuspended in 200 μL extraction buffer (Triton 100X 2%, 100 mM Tris–HCl pH 8.5, 100 mM NaCl, 10 mM EDTA pH 8.0, 1% sodium dodecyl sulphate, 200 μL of phenol-chloroform-isoamilic alcohol and 0.5 mL of 0.5 mm diameter glass beads). Samples were bead-beaten on a vortex for 2 minutes and centrifuged for 5 minutes at 14,000 rpm. The DNA was precipitated from the aqueous phase by adding 0.5 volumes of ethanol, followed by centrifugation at 14000 rpm for 3 min in a micro centrifuge. The pellet was washed with 70% ethanol and resuspended in 400 μL of TE buffer (10 mM Tris–HCl, 1 mM EDTA pH 7.5) with 3 μg RNase, and then incubated at 37 °C for 30 min.

The D1/D2 domain at the 5′ end of the rRNA gene was according to Guadet et al (1989) [44] was symmetrically amplified with primers NL-1 (5′- GCATATCAATAAGCGGAGGAAAAG) and NL-4 (5′-GGTCCGTGTTTCAAGACGG) described previously by Kurtzman and Robnett (1998) [43]. The amplification was done in a thermocycler (GenAmp 9700, Applied Biosystems) for 36 PCR cycles with annealing at 52 °C, extension at 72 °C for 2 minutes, and denaturation at 94 °C for 1 minute. The PCR product DNA was purified with Geneclean II (Bio 101, La Jolla, California) according to the manufacturer’s instructions. The amplified DNA was visualized through electrophoresis in 1.5% agarose in 1X TAE buffer (0.04 M Trisacetate, 1 mM EDTA pH 8.0) and stained with ethidium bromide (8 × 10^− 5^ μg/mL). Both strands of the rDNA region were sequenced using a BigDye Terminator Cycle sequencing kit (Applied Biosystems Inc., Foster City, California). Sequence data was aligned to obtain a consensus sequence using the software Multiseq (Lasergene). Existing sequences from other type strains were retrieved from GenBank for alignment.

### Fermenter studies of Pseudozyma hubeiensis BOT-O, *Rhodosporidium toruloides* BOT-A2 and *Rhodotorula glutinis* CCUG32821

Three strains were selected for studies in fermenter settings using 2 l Infors reactors. The two strains *P. hubeiensis* BOT-O and *R. toruloides* BOT-A2 were selected based on their performance in the screenings. The working volumes were 1 L YNB (without ammonium sulphate) media containing xylose (40 g/L), ammonium sulphate (1.13 g/L), and yeast extract (1 g/L), buffered to pH 5.5 with a succinate buffer. About 1 ml of PEG antifoam was added to 1 L of medium. The stirring was set to 700 rpm, temperature to 30 °C, aeration to 60% O_2_ and the pH was set to pH 5.5 using 1 M solutions of HCl and NaOH. Growth, xylose consumption and fatty acid accumulation was monitored in duplicates during cultivation.

### Comparative C/N 80 shake flask studies of strain BOT-O and BOT-A2 on glucose and xylose

Two strains were selected to study the effects of different carbon sources on growth, sugar and nitrogen utilisation, and lipid accumulation. Working volume was 125 mL of YNB (without ammonium sulphate) medium containing 2% of either glucose or xylose as the carbon source and ammonium sulphate to a C/N ratio of 80 g/g in 500 mL shake flasks. The medium was buffered to pH 5.5 with a 1 M potassium buffer (118.3 g/L KH_2_PO_4_ and 22.99 g/L K_2_HPO_4_). Cultures were inoculated at OD_600nm_ 0.1 from an overnight culture in YPD. Shake flasks were shaken at 210 rpm for approximately 56 h at 30 °C. Cultivations were done in triplicates. Samples were taken regularly to measure growth, carbon and nitrogen consumption, as well as lipid accumulation.

### Xylose analysis on HPLC

Xylose analysis was performed by HPLC (Merck Hitachi, Japan L6200A Intelligent pumps) with a CMA/200 auto sampler (Solna, Sweden) was used. The detector was an electrochemical detector ED40 (Ca, USA) and the columns were Dionex PA1 anionic exchange columns. The eluent used was NaOH (10 mM) at a flow of 0.6 mL/min. Samples were analyzed using Borwin software (France) and the concentration was calculated based on an external standard curve.

### Nitrogen consumption measurements

The consumption of nitrogen was measured in triplicates using the Ammonia Assay Kit (Rapid) (Megazyme Ltd., Bray, Ireland) at half volumes and according to the manufacturer’s protocol. Optical density at 340 nm was assessed using a Genesys 20 spectrophotometer (Thermo Fisher Scientific Inc., Waltham, MA, USA).

### Statistical analysis

Experiments were performed in duplicates (fermenter studies) or triplicates (shake flask cultivations), with the exception of the screening. All data are expressed as arithmetic means with standard deviations.

## Supplementary Information

**Additional file 1: Supplemental Figure 1.** Fatty acid (FA) composition and total FA content (%) per dry weight biomass for tested strains after cultivation in five different media. The media were: YNB with either glucose (called Glucose), xylose (called Xylose), or glycerol (called Glycerol), molasses with or without phosphate addition (phosphate addition is indicated by P next to the strain name), and enzymatically treated wheat hydrolysate diluted to 25% of the original concentration (called EWH 25%). The fatty acids detected were 14:0, 16:0, 16:1, 18:0, 18:1, 18:2, 18:3, 20:0, 22:0, 22:2 and 24:0. The detailed corresponding data can be found in Supplementary Table S1. Strains with poor growth were not examined due to too little biomass formation.

**Additional file 2: Supplementary Table S1.** Total fatty acid content (given as % of dry weight biomass) and the fatty acid composition (given as mg/g dry weight biomass) in different yeast isolates after cultivation in various substrates. Some isolates did not grow in all media, hence data of fatty acid content or profile is not reported for all combinations of isolates and media.

**Additional file 3: Supplementary Table S2.** Yeast isolates species and the GenBank code number for the identified strains.

## Data Availability

All data generated and analysed during this study are included in this published article and its supplementary files. 18S DNA sequences from identified strains were deposited in GenBank with the accession numbers ON644555 for *S. salmonicolor* BOT-10.3, ON644556 for *P. hubeiensis* BOT-O, ON644557 for *R. toruloides* BOT-A2, ON644558 for *R. minuta* BOT-1, ON644559 for *C. flavescens* BOT-6.1, ON644560 for *R. minuta* BOT-6.2, ON644561 for *R. oligophage* BOT-J.1, ON644562 for *R. glutinis* BOT-4, and ON644563 for *R. toruloides* BOT-8. Isolated strains from this work are available from the corresponding author upon request.
