# Supplementary material for: Screening of xylose utilizing and high lipid producing yeast strains as a potential candidate for industrial application
Source: BMC Microbiol. 2022 Jul 7;22:173. doi: 10.1186/s12866-022-02586-y (PMC9261059; doi:10.1186/s12866-022-02586-y)
Supplement: Supplementary file 1 — Additional file 1: Supplemental Figure 1. Fatty acid (FA) composition and total FA content (%) per dry weight biomass for tested strains after cultivation in five different media. The media were: YNB with either glucose (called Glucose), xylose (called Xylose), or glycerol (called Glycerol), molasses with or without phosphate addition (phosphate addition is indicated by P next to the strain name), and enzymatically treated wheat hydrolysate diluted to 25% of the original concentration (called EWH 25%). The fatty acids detected were 14:0, 16:0, 16:1, 18:0, 18:1, 18:2, 18:3, 20:0, 22:0, 22:2 and 24:0. The detailed corresponding data can be found in Supplementary Table S1. Strains with poor growth were not examined due to too little biomass formation. [file 12866_2022_2586_MOESM1_ESM.docx]

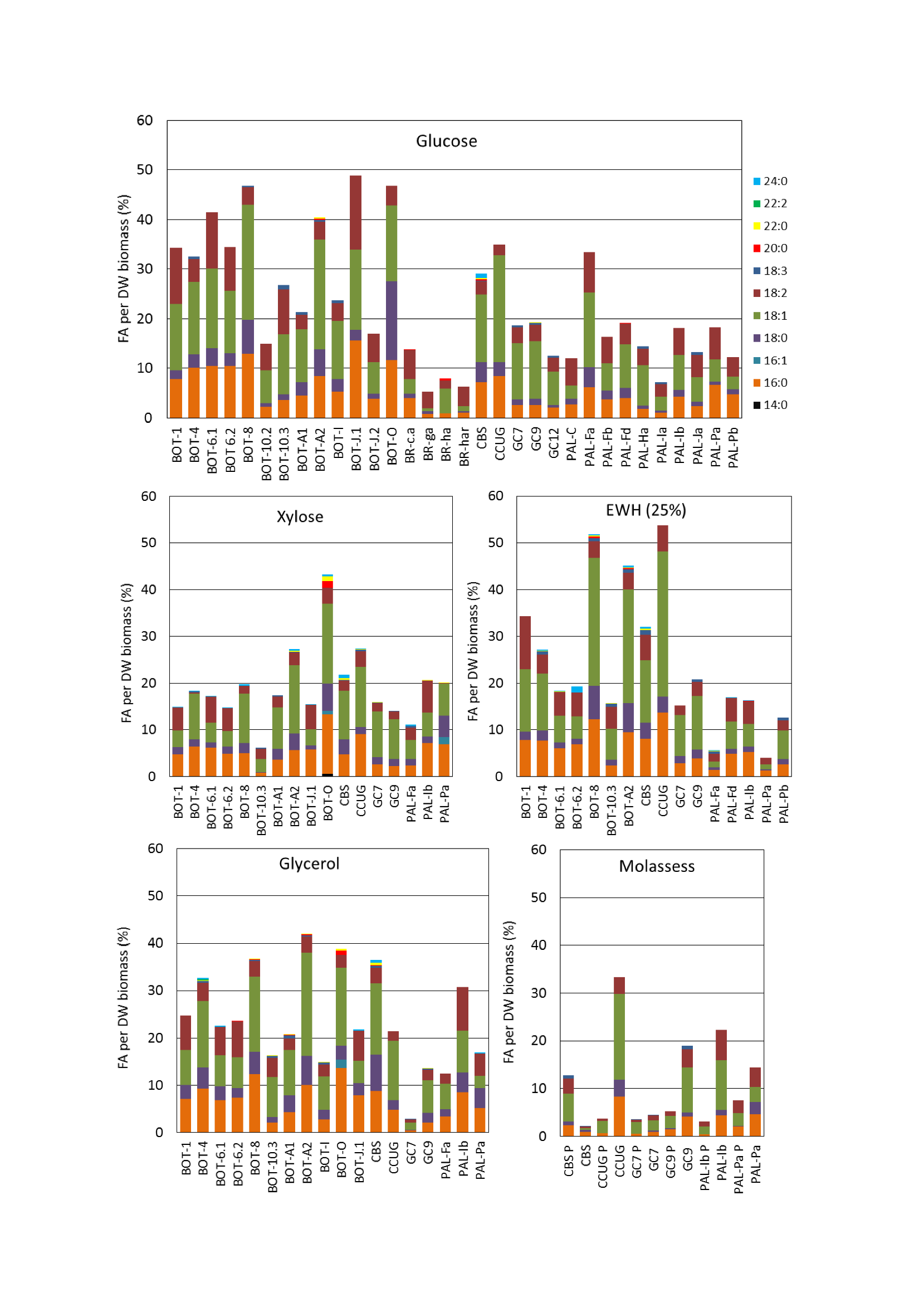


**Supplemental Figure 1**. Fatty acid (FA) composition and total FA content (%) per dry weight biomass for tested strains after cultivation in five different media. The media were: YNB with either glucose (called Glucose), xylose (called Xylose), or glycerol (called Glycerol), molasses with or without phosphate addition (phosphate addition is indicated by **P** next to the strain name), and enzymatically treated wheat hydrolysate diluted to 25% of the original concentration (called EWH 25%). The fatty acids detected were 14:0, 16:0, 16:1, 18:0, 18:1, 18:2, 18:3, 20:0, 22:0, 22:2 and 24:0. The detailed corresponding data can be found in Supplementary Table S1. Strains with poor growth were not examined due to too little biomass formation.
