# Supplementary material for: Screening of xylose utilizing and high lipid producing yeast strains as a potential candidate for industrial application
Source: BMC Microbiol. 2022 Jul 7;22:173. doi: 10.1186/s12866-022-02586-y (PMC9261059; doi:10.1186/s12866-022-02586-y)
Supplement: Supplementary file 2 — Additional file 2: Supplementary Table S1. Total fatty acid content (given as % of dry weight biomass) and the fatty acid composition (given as mg/g dry weight biomass) in different yeast isolates after cultivation in various substrates. Some isolates did not grow in all media, hence data of fatty acid content or profile is not reported for all combinations of isolates and media. [file 12866_2022_2586_MOESM2_ESM.docx]

**Supplementary table S1**. Total fatty acid content (given as % of dry weight biomass) and the fatty acid composition (given as mg/g dry weight biomass) in different yeast isolates after cultivation in various substrates. Some isolates did not grow in all media, hence data of fatty acid content or profile is not reported for all combinations of isolates and media.

|  |  | **Total FA (% of DW)** | **Fatty acid per dry weight biomass (mg/g)** | | | | | | | | | | |
| --- | --- | --- | --- | --- | --- | --- | --- | --- | --- | --- | --- | --- | --- |
| **Strain** | **Substrate** |  | **14:0** | **16:0** | **16:1** | **18:0** | **18:1** | **18:2** | **18:3** | **20:0** | **22:0** | **22:2** | **24:0** |
| PAL-Fa | Glucose | **34,3** | 0 | 77,9 | 0 | 18,37 | 132,9 | 113,7 | 0 | 0 | 0 | 0 | 0 |
|  | Xylose | **11,1** | 0 | 23,3 | 0 | 14,3 | 41,2 | 26,7 | 2,8 | 0 | 0 | 0 | 3,0 |
|  | Glycerol | **12,5** | 0 | 33,3 | 0 | 16,0 | 54,1 | 21,9 | 0 | 0 | 0 | 0 | 0 |
|  |  |  |  |  |  |  |  |  |  |  |  |  |  |
|  | EWH | **5,6** | 0 | 14,6 | 0 | 5,4 | 12,4 | 16,6 | 4,3 | 0,5 | 1,0 | 0 | 1,0 |
| PAL-Fb | Glucose | **16,3** | 0 | 36,9 | 0 | 18,7 | 55,0 | 52,4 | 0 | 0 | 0 | 0 | 0 |
| PAL-Fd | Glucose | **19,1** | 0 | 40,0 | 0 | 20,2 | 87,6 | 42,0 | 0 | 1 | 0 | 0 | 0 |
|  | EWH | **17,0** | 0 | 48,8 | 0 | 10,2 | 58,7 | 20,7 | 0 | 0,5 | 0 | 0 | 1,2 |
| PAL-Ia | Glucose | **7,2** | 0 | 11,0 | 0 | 3,5 | 28,6 | 25,4 | 3,7 | 0 | 0 | 0 | 0 |
| PAL-Ib | Glucose | **18,2** | 1,0 | 42,0 | 0 | 13,8 | 70,3 | 54,5 | 0 | 0 | 0 | 0 | 0 |
|  | Xylose | **20,6** | 0 | 72,2 | 0 | 13,1 | 52,0 | 67,6 | 0 | 0,5 | 0,5 | 0 | 0 |
|  | Glycerol | **30,8** | 0 | 84,7 | 0 | 42,1 | 88,3 | 93,2 | 0 | 0 | 0 | 0 | 0 |
|  | EWH | **16,3** | 0 | 52,9 | 0 | 10,9 | 48,2 | 49,2 | 0 | 0,5 | 0,5 | 0 | 1,3 |
|  | Molasses + P | **3,1** | 0 | 4,4 | 0 | 0,2 | 15,6 | 10,3 | 0 | 0 | 0 | 0 | 0 |
|  | Molasses – P | **22,4** | 0 | 43,1 | 0 | 11,5 | 105,4 | 63,6 | 0 | 0 | 0 | 0 | 0 |
| PAL-Ja | Glucose | **13,3** | 0 | 23,8 | 0 | 8,2 | 49,6 | 45,0 | 6,5 | 0 | 0 | 0 | 0 |
| PAL-Ha | Glucose | **14,5** | 0 | 17,7 | 0 | 6,5 | 82,1 | 32,4 | 6,0 | 0 | 0 | 0 | 0 |
| PAL-Pa | Glucose | **18,2** | 0 | 66,6 | 0 | 7,0 | 43,3 | 65,1 | 0 | 0 | 0 | 0 | 0 |
|  | Xylose | **20,0** | 0 | 68,9 | 16 | 45,5 | 68,6 | 0,8 | 0,3 | 0 | 0,2 | 0 | 0 |
|  | Glycerol | **16,9** | 0 | 51,4 | 0 | 42,0 | 26,4 | 46,6 | 0 | 1,0 | 0 | 0 | 2,0 |
|  | EWH | **4,0** | 0 | 13,5 | 0 | 1,7 | 10,4 | 14,5 | 0 | 0 | 0 | 0 | 0 |
|  | Molasses + P | **7,5** | 0 | 21,4 | 0 | 0,7 | 26,5 | 26,3 | 0 | 0 | 0 | 0 | 0 |
|  | Molasses – P | **14,4** | 0 | 45,8 | 0 | 25,4 | 31,8 | 41,3 | 0 | 0 | 0 | 0 | 0 |
| PAL-Pb | Glucose | **12,3** | 0 | 47,9 | 0 | 9,4 | 25,9 | 39,7 | 0 | 0 | 0 | 0 | 0 |
|  | EWH | **12,6** | 0 | 26,3 | 0 | 11,4 | 60,0 | 21,7 | 6,6 | 0 | 0 | 0 | 0 |
| PAL-C | Glucose | **12,0** | 0 | 27,4 | 0 | 11,1 | 26,5 | 55,0 | 0 | 0 | 0 | 0 | 0 |
| BOT-10.2 | Glucose | **15,0** | 0 | 22,1 | 0 | 7,0 | 67,3 | 53,4 | 0 | 0 | 0 | 0 | 0 |
| BOT-10.3 | Glucose | **26,8** | 0 | 35,7 | 0 | 12,1 | 120,6 | 90,6 | 9,2 | 0 | 0 | 0 | 0 |
|  | Xylose | **6,2** | 0 | 7,7 | 0 | 1,7 | 27,6 | 22,2 | 2,8 | 0 | 0 | 0 | 0 |
|  | Glycerol | **16,3** | 0 | 21,3 | 0 | 11,4 | 84,7 | 40,7 | 3,4 | 0 | 1,8 | 0 | 0 |
|  | EWH | **15,7** | 0 | 23,2 | 0 | 12,4 | 66,1 | 47,7 | 5,7 | 0,7 | 1,6 | 0 | 0 |
| BOT-O | Glucose | **46,8** | 0 | 115,8 | 0 | 159,9 | 152,5 | 39,38 | 0 | 0 | 0 | 0 | 0 |
|  | Xylose | **43,2** | 5,2 | 127,4 | 8,4 | 58,53 | 170,1 | 34,8 | 0 | 13,2 | 10,6 | 0 | 3,8 |
|  | Glycerol | **38,9** | 0 | 136,3 | 17,7 | 29,6 | 165,2 | 26,6 | 0 | 9,9 | 3,7 | 0 | 0 |
| BOT-A1 | Glucose | **21,3** | 0 | 45,7 | 0 | 25,8 | 107,4 | 29,6 | 4,8 | 0 | 0 | 0 | 0 |
|  | Xylose | **27,3** | 0 | 57,1 | 0 | 35,2 | 146,9 | 26,9 | 2 | 0 | 1,9 | 0 | 3,6 |
|  | Glycerol | **20,8** | 0 | 42,9 | 0 | 35,2 | 96,4 | 52,2 | 5,8 | 1,1 | 1,1 | 0 | 0 |
| BOT-A2 | Glucose | **40,4** | 0 | 84,4 | 0 | 53,8 | 221,5 | 35,8 | 3,7 | 2,2 | 2,3 | 0 | 0 |
|  | Xylose | **27,3** | 0 | 57,1 | 0 | 35,2 | 145,9 | 26,9 | 2,0 | 0 | 1,9 | 0 | 3,6 |
|  | Glycerol | **42,1** | 0 | 100,6 | 0 | 60,7 | 219,2 | 33,5 | 3,3 | 1,7 | 1,7 | 0 | 0 |
|  | EWH | **45,1** | 0 | 94,8 | 0 | 62,0 | 243,5 | 35,7 | 7,2 | 2,3 | 2,3 | 0 | 3,1 |
| BOT-I | Glucose | **23,7** | 0 | 53,0 | 0 | 25,5 | 116,5 | 35,5 | 6,4 | 0 | 0 | 0 | 0 |
|  | Glycerol | **14,8** | 0 | 27,9 | 0 | 20,5 | 69,5 | 26,1 | 3,5 | 0 | 0,9 | 0 | 0 |
| BOT-1 | Glucose | **34,0** | 0 | 77,9 | 0 | 18,4 | 132,9 | 113,7 | 0 | 0 | 0 | 0 | 0 |
|  | Xylose | **15,0** | 0 | 47,6 | 0 | 14,6 | 36,0 | 49,9 | 0 | 0 | 0,6 | 0 | 0,9 |
|  | Glycerol | **24,7** | 0 | 71,5 | 0 | 29,4 | 73,6 | 72,8 | 0 | 0 | 0 | 0 | 0 |
|  | EWH | **34,3** | 0 | 77,9 | 0 | 18,4 | 132,9 | 113,7 | 0 | 0 | 0 | 0 | 0 |
| BOT-6.1 | Glucose | **41,4** | 0 | 104,3 | 0 | 36,6 | 160,4 | 113,4 | 0 | 0 | 0 | 0 | 0 |
|  | Xylose | **17,1** | 0 | 61,7 | 0 | 11,8 | 41,0 | 56,1 | 0 | 0 | 0 | 0 | 0,7 |
|  | Glycerol | **22,5** | 0 | 68,4 | 0 | 30,1 | 64,7 | 59,5 | 0 | 1,0 | 0 | 0 | 1,6 |
|  | EWH | **18,3** | 0 | 60,3 | 0 | 13,0 | 56,6 | 51,2 | 0 | 0,6 | 0,5 | 0 | 1,3 |
| BOT-6.2 | Glucose | **34,4** | 0 | 104,7 | 0 | 25,4 | 125,9 | 88,4 | 0 | 0 | 0 | 0 | 0 |
|  | Xylose | **14,8** | 0 | 49,4 | 0 | 15,4 | 32,8 | 49,7 | 0 | 0 | 0 | 0 | 1,2 |
|  | Glycerol | **23,5** | 0 | 73,67 | 0 | 20,8 | 64,3 | 75,5 | 0 | 0,9 | 0 | 0 | 0 |
|  | EWH | **19,3** | 0 | 68,7 | 0 | 11,9 | 49,0 | 50,4 | 0 | 0 | 0 | 0 | 13,0 |
| BOT-J.1 | Glucose | **49** | 0 | 155,3 | 0 | 22,3 | 161,8 | 148,7 | 0 | 0 | 0 | 0 | 0 |
|  | Xylose | **15,4** | 0 | 57,6 | 0 | 9,4 | 34,0 | 51,7 | 0 | 0 | 0 | 0 | 1,5 |
|  | Glycerol | **21,8** | 0 | 78,9 | 0 | 25,1 | 47,1 | 64,5 | 0,3 | 0 | 0 | 0 | 1,8 |
| BOT-J.2 | Glucose | **17,0** | 0 | 38,2 | 0 | 10,3 | 64,2 | 57,3 | 0 | 0 | 0 | 0 | 0 |
| BOT-4 | Glucose | **32,5** | 0 | 101,1 | 0 | 26,7 | 146,7 | 45,7 | 4,5 | 0 | 0 | 0 | 0 |
|  | Xylose | **18,4** | 0 | 64,2 | 0 | 15,0 | 98,4 | 2,9 | 2,0 | 0 | 0 | 0 | 1,8 |
|  | Glycerol | **32,7** | 0 | 92,5 | 0 | 45,0 | 140,3 | 38,7 | 3,6 | 0 | 2,3 | 2,3 | 2,3 |
|  | EWH | **27,2** | 0 | 76,4 | 0 | 21,9 | 122,0 | 41,5 | 5,9 | 0 | 1,8 | 0 | 2,1 |
| BOT-8 | Glucose | **46,8** | 0 | 129,2 | 0 | 68,5 | 231,8 | 35,5 | 3,1 | 0 | 0 | 0 | 0 |
|  | Xylose | **19,7** | 0 | 49,9 | 0 | 22,5 | 104,6 | 16,9 | 0 | 0 | 0 | 0 | 3,5 |
|  | Glycerol | **36,8** | 0 | 123,3 | 0 | 47,3 | 159,2 | 33,0 | 2,2 | 1,2 | 1,6 | 0 | 0 |
|  | EWH | **51,8** | 0 | 122,4 | 0 | 72,0 | 273,0 | 35,5 | 7,1 | 3,4 | 2,8 | 0 | 2,0 |
| GC-7 | Glucose | **18,5** | 0 | 26,2 | 0 | 11,6 | 113,4 | 30,8 | 4,3 | 0 | 0 | 0 | 0 |
|  | Xylose | **16,0** | 0 | 25,7 | 0 | 15,3 | 98,2 | 17,5 | 1,5 | 0,6 | 1,3 | 0 | 0 |
|  | Glycerol | **2,7** | 0 | 1,0 | 0 | 1,1 | 15,7 | 5,9 | 0,4 | 0 | 0 | 0 | 0 |
|  | EWH | **15,2** | 0 | 28,8 | 0 | 14,2 | 88,8 | 20,2 | 0 | 0 | 0 | 0 | 0 |
|  | Molasses + P | **3,5** | 0 | 5,3 | 0 | 0,6 | 23,6 | 5,2 | 0,5 | 0 | 0 | 0 | 0 |
|  | Molasses - P | **4,5** | 0 | 9,8 | 0 | 1,7 | 22,2 | 9,8 | 1,6 | 0 | 0 | 0 | 0 |
| GC-9 | Glucose | **19,3** | 0 | 25,3 | 0,9 | 11,8 | 116,1 | 34,1 | 3,4 | 0 | 1,0 | 0 | 0 |
|  | Xylose | **14,0** | 0 | 22,6 | 0 | 15,2 | 84,3 | 16,9 | 1,5 | 0 | 0 | 0 | 0 |
|  | Glycerol | **13,6** | 0 | 20,6 | 0 | 21,3 | 69,3 | 21,3 | 2,5 | 0 | 1,2 | 0 | 0 |
|  | EWH | **20,8** | 0 | 38,0 | 0 | 19,4 | 114,2 | 30,6 | 5,8 | 0 | 0 | 0 | 0 |
|  | Molasses + P | **5,3** | 0 | 14,2 | 0 | 2,7 | 26,2 | 9,9 | 0 | 0 | 0 | 0 | 0 |
|  | Molasses – P | **18,9** | 0 | 40,7 | 0 | 9,7 | 93,4 | 38,5 | 7,2 | 0 | 0 | 0 | 0 |
| GC-12 | Glucose | **12,6** | 0 | 20,4 | 0 | 5,9 | 66,6 | 28,0 | 4,6 | 0 | 0 | 0 | 0 |
| BR-c.a | Glucose | **13,6** | 0 | 40,2 | 0,3 | 8,5 | 29,0 | 58,2 | 0 | 0,2 | 0 | 0 | 0 |
| BR-g.a | Glucose | **5,3** | 0 | 8,4 | 0 | 4,5 | 6,8 | 33,3 | 0 | 0 | 0 | 0 | 0 |
| BR-h.a | Glucose | **8,0** | 0 | 8,8 | 0 | 1,0 | 49,0 | 16,5 | 0,7 | 3,6 | 0 | 0 | 0 |
| BR-h.ar | Glucose | **6,3** | 0 | 102 | 0 | 2,7 | 10,6 | 39,3 | 0 | 0 | 0 | 0 | 0 |
| CBS14 | Glucose | **29,1** | 0 | 72,2 | 0 | 40,5 | 135,8 | 26,6 | 2,3 | 1,5 | 3,2 | 0 | 8,9 |
|  | Xylose | **21,8** | 0 | 48,1 | 0 | 31,3 | 104,9 | 21,0 | 2,0 | 0 | 2,7 | 0 | 7,8 |
|  | Glycerol | **36,5** | 0 | 88,2 | 0 | 75,8 | 151,6 | 33,6 | 2,9 | 2,4 | 4,1 | 0 | 6,9 |
|  | EWH | **32,0** | 0 | 80,6 | 0 | 34,3 | 134,2 | 53,9 | 10,7 | 0 | 2,2 | 0 | 4,3 |
|  | Molasses + P | **12,8** | 0 | 23,6 | 0 | 7,0 | 58,4 | 32,2 | 6,9 | 0 | 0 | 0 | 0 |
|  | Molasses – P | **2,3** | 0 | 9,2 | 0 | 3,8 | 4,2 | 4,0 | 1,5 | 0 | 0 | 0 | 0 |
| CCUG 32821 | Glucose | **35,0** | 0 | 84,6 | 0 | 28,1 | 215,6 | 21,7 | 0 | 0 | 0 | 0 | 0 |
|  | Xylose | **27,4** | 0 | 90,8 | 0 | 15,1 | 128,9 | 33,7 | 2,9 | 0,7 | 1,4 | 0 | 1,0 |
|  | Glycerol | **21,4** | 0 | 48,4 | 0 | 20,5 | 124,3 | 20,9 | 0 | 0 | 0 | 0 | 0 |
|  | EWH | **53,8** | 0 | 137,1 | 0 | 33,3 | 311,3 | 56,3 | 0 | 0 | 0 | 0 | 0 |
|  | Molasses + P | **3,7** | 0 | 6,6 | 0 | 0,4 | 25,2 | 5,1 | 0 | 0 | 0 | 0 | 0 |
|  | Molasses – P | **33,4** | 0 | 83,2 | 0 | 35,6 | 178,8 | 36,0 | 0 | 0 | 0 | 0 | 0 |
