# Supplementary material for: Screening of xylose utilizing and high lipid producing yeast strains as a potential candidate for industrial application
Source: BMC Microbiol. 2022 Jul 7;22:173. doi: 10.1186/s12866-022-02586-y (PMC9261059; doi:10.1186/s12866-022-02586-y)
Supplement: Supplementary file 3 — Additional file 3: Supplementary Table S2. Yeast isolates species and the GenBank code number for the identified strains. [file 12866_2022_2586_MOESM3_ESM.docx]

| Strain | Species | GenBank code |
| --- | --- | --- |
| BOT-10.3 | *Sporidiobolus salmonicolor* | ON644555 |
| BOT-O | *Pseudozyma hubeiensis* | ON644556 |
| BOT-A2 | *Rhodosporidium toruloides* | ON644557 |
| BOT-1 | *Rhodotorula minuta* | ON644558 |
| BOT-6.1 | *Cryptococcus flavescens* | ON644559 |
| BOT-6.2 | *Rhodotorula minuta* | ON644560 |
| BOT-J.1 | *Rhodotorula oligophage* | ON644561 |
| BOT-4 | *Rhodotorula glutinis / Rhodosporidium diobovatum* | ON644562 |
| BOT-8 | *Rhodosporidium toruloides* | ON644563 |

Supplementary table S2. Yeast isolates species and the GenBank code number for the identified strains.
